# Intox - A Prospective Measurement of Blood Concentrations of Routine Drugs in Patients Treated in the Intensive Care Unit

**DOI:** 10.1186/2197-425X-3-S1-A496

**Published:** 2015-10-01

**Authors:** U Lennborn, E Nielsen, H Sandler, M Bertilsson, A Johansson, J Ahlner, FC Kugelberg, S Rubertsson

**Affiliations:** Uppsala University, Department of Surgical Sciences / Anaesthesiology and Intensive Care Medicine, Uppsala, Sweden; Uppsala University, Department of Pharmaceutical Biosciences, Uppsala, Sweden; Uppsala University, Department of Surgical Sciences / Forensic Medicine, Uppsala, Sweden; Uppsala University, Uppsala Clinical Research Center, Uppsala, Sweden; National Board of Forensic Medicine, Department of Forensic Genetics and Forensic Toxicology, Linköping, Sweden

## Introduction

Standard dosages and resulting range of blood concentrations of analgesic and sedative drugs given to ICU patients, with a varying degree of organ failure, may affect the disposition (absorption, distribution, metabolism and excretion) of the drugs administered. Drug metabolism in ICU patients need to be further investigated.

## Objectives

The aim of this project is to study the pharmacokinetics of analgesic and sedative drugs in a cohort of a Swedish general ICU with special focus on fentanyl and propofol.

## Methods

A prospective, observational pilot study of 69 patients was performed in the general ICU at Uppsala University hospital, Sweden. Blood samples were collected according to routine procedures upon the patient's arrival at the ICU, and then twice daily. the blood samples were sent to the National Board of Forensic Medicine in Linköping, Sweden, for analysis of drug concentrations.

## Results

**Figure 1:**
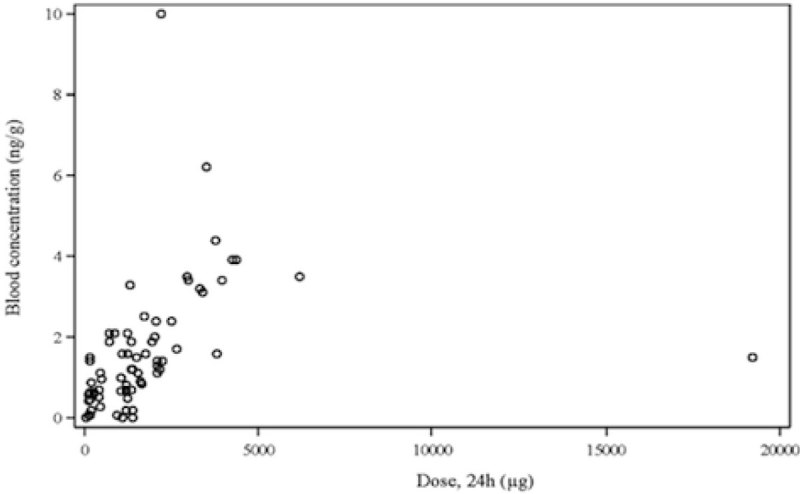
***Fentanyl.***

**Figure 2:**
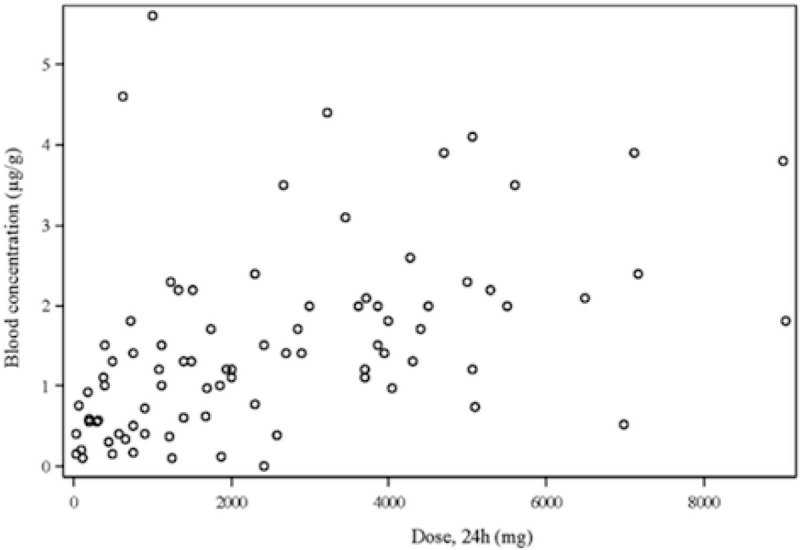
***Propofol.***

Measured blood concentrations related to the total 24-hour dose for fentanyl is showed in Figure 1 and for propofol in Figure 2.

## Conclusions

The variety of drug concentrations observed in relation to standard dosages of fentanyl and propofol, together with other drugs, need to be further investigated in a larger ICU population since this may affect the length of stay in the ICU and in the end health economics.

